# Fundus fluorescence Angiography in diagnosing diabetic retinopathy

**DOI:** 10.12669/pjms.336.13405

**Published:** 2017

**Authors:** Shuhui Wang, Yuqin Zuo, Ning Wang, Bin Tong

**Affiliations:** 1 Shuhui Wang, Department of Ophthalmology, Binzhou People’s Hospital, Shandong, 256600, China; 2 Yuqin Zuo, Department of GI Medicine, Binzhou People’s Hospital, Shandong, 256600, China; 3 Ning Wang, Department of Psychiatry, Binzhou People’s Hospital, Shandong, 256600, China; 4 Bin Tong, Department of Ophthalmology, Binzhou People’s Hospital, Shandong, 256600, China

**Keywords:** Diabetic retinopathy, Diagnosis, Fundus fluorescence angiography

## Abstract

**Objective:**

To investigate the manifestation characteristics of fundus fluorescence angiography (FFA) and its values in diagnosing diabetic retinopathy through comparing direct ophthalmoscopy.

**Methods:**

Two hundred fifty patients (500 eyes) who were suspected as diabetic retinopathy and admitted to the hospital between February 2015 and December 2016 were selected. They underwent direct ophthalmoscopy and FFA. The manifestation characteristics of FFA in the diagnosis of diabetic retinopathy were summarized. The two examination methods were compared.

**Results:**

In the diagnosis with direct ophthalmoscopy, 375 eyes out of 500 eyes were diagnosed as diabetic retinopathy (75%); there were 74 eyes at stage I, 88 eyes at stage II, 92 eyes at stage III, 83 eyes of stage IV, 28 eyes of stage V and 10 eyes of stage VI. In the diagnosis with FFA, 465 eyes out of 500 eyes were diagnosed as diabetic retinopathy (93%); there were 94 eyes at stage I, 110 eyes at stage II, 112 at stage III, 92 eyes at stage IV, 41 eyes at stage V and 16 eyes at stage VI. The detection rate of diabetic retinopathy using FFA was significantly higher than that using direct ophthalmoscopy (P<0.05). FFA found that 316 eyes had non-proliferative retinopathy (67.96%), 75 eyes had pre-proliferative lesions (16.13%), 149 eyes had proliferative lesions (32.04%), 135 eyes had diabetic maculopathy (29.03%) and 31 eyes had diabetic optic disc lesions (6.67%).

**Conclusion:**

The detection rate of diabetic retinopathy using FFA is higher than that using direct ophthalmoscopy. FFA could accurately determine clinical stage. Therefore, it is an important approach in treatment efficacy evaluation and treatment guidance, suggesting a significant application value.

## INTRODUCTION

Diabetics with a high disability rate and fatality rate has been a common disease affecting the health of people in modern society.^1^ Diabetics is manifested as changes of small vessels and microvessels. Diabetic retinopathy is one of the severest complications in diabetic microangiopathy and the main cause for the blindness of diabetic patients.^2^,^3^ But diabetic retinopathy is difficult to be discovered in the early stage, leading to delayed treatment. It was reported that the incidence of diabetic retinopathy among patients who have had diabetes for five years was between 49% and 58%.^4^ Therefore, early diagnosis of diabetic retinopathy is of great significance to clinical treatment and the improvement of prognosis.

Direct ophthalmoscopy is still an important diagnostic approach in the diagnosis of diabetic retinopathy. With the popularization of fundus fluorescence angiography (FFA), the position of diabetic retinopathy in the diagnosis of diabetic retinopathy is gradually highlighted. FFA is usually used for examining fundus oculi disease. Using FFA, patients were intravenously injected with fluorescent substances, sodium fluorescein usually. The fluorescent substances flow to fundus vessels through blood circulation. Then the fluorescent substances were simulated with blue light to send out yellow-green fluorescence. Finally fundus photograph was performed under special optical filter. Photograph lasted in the whole process of fundus blood circulation as fundus images at different time points and parts are needed for experimental analysis.^5^ There are lots of studies concerning FFA. Cecilia S et al. found that the function of FFA was stronger than spectral domain optical coherence tomography (SDOCT) in the evaluation of Immuno Radio Metric Assay (IRMA) and neovascularization elsewhere (NVE).^6^ Wang Yongcheng et al. found FFA could discover various microvascular damages and the damage degree and retinal ischemia in the early stage and played an important role in the diagnosis of diabetic optic neuropathy.^7^ But few studies concerned the values of FFA and direct ophthalmoscopy in the diagnosis of diabetic retinopathy. This study carried out fundus examination on 500 eyes which were suspected as diabetic retinopathy using FFA and direct ophthalmoscopy and compared the examination results.

## METHODS

### General data

Two hundred and fifty patients with diabetic retinopathy who were admitted to the Binzhou People’s Hospital, Shandong, China between February 2015 and December 2016 were selected as research subjects. All the patients conformed to the international diagnostic criteria of diabetic retinopathy (2003).^8^ Those who were not suitable for examination because of conjunctival and corneal inflammation, suffered from internal ophthalmopathy, eye traumas and intraocular surgery previously, or had other eye diseases such as cataract, glaucoma, optic nerve disease, retinal detachment and uveitis were excluded. There were 145 males and 105 females; they aged from 34 years to 84 years (average 54.32±3.22 years). Among 250 patients, 63 patients were no younger than 60 years, 88 patients were aged between 50 years and 59 years, 67 patients were aged between 40 years and 49 years, and 32 patients were aged between 30 years and 39 years. The patients have had diabetes for two months to 28 years (average 8.72±2.37 years). Among 250 patients, 58 patients have had diabetes for no less than 10 years, 110 patients have had diabetes for 5 to 9 years, and 82 patients have had diabetes for no more than 5 years. All the included patients signed informed consent, and the study has been approved by ethics committee of the Binzhou People’s Hospital, Shandong, China.

### Research methods

All the patients underwent vision, slit-lamp and intraocular pressure examination. After full mydriasis using compound tropicamide, fundus examination was performed and the results were recorded.

### Direct ophthalmoscopy

The optic disc was examined by direct ophthalmoscopy. Then the peripheral part of the retina was examined following blood vessels. Finally, the macular area was examined.

### FFA

After the absence of obvious contraindications and negative fluorescein sodium skin test were determined by general examination for blood pressure, pulse, electrocardiogram and biochemistry, 5 mL of fluorescein sodium (20%) was intravenously injected for 5 s. One eye with poor visual function was determined as the mainly illustrated eye; the two eyes were alternately photographed; five to seven fields were selected and photographed discontinuously after 1 min of venous filling. Radiography lasted for 10 to 15 min. The examinations aforementioned were carried out by the same doctor. The duration of different stages of radiography was strictly controlled, which was beneficial to the accuracy of examination.

### Observation record of retinopathy

The amount of microangioma, category of exudation and presence of new vessels, fibrovascular proliferation and retinal detachment or not were observed. The number of cases of lesions detected by the two examination methods and corresponding stages were recorded. The manifestation characteristics of diabetic retinopathy in FFA were concluded.

### Staging criteria of diabetic retinopathy

Criteria of six stages of diabetic retinopathy formulated by the National Academic Conference of Fundus Diseases (1985) were as follows.^9^ No obvious retinopathy and small hemorrhagic spots at posterior pole were determined as stage I. Scattered dotted hyperfluorescence and presence of microangioma were determined as stage II. Scattered dotted hyperfluorescence and more microangioma than stage II were determined as stage III. Presence of new vessels in fundus or hemorrhagic symptoms in vitreous body was determined as stage IV. Presence of new vessels in fundus and fibroblast proliferation were determined as stage V. Tractional retinal detachment in addition to the symptoms of stage V was determined as stage VI. Diabetic retinopathy at stage I, II and III was non-proliferative diabetic retinopathy, while diabetic retinopathy at stage IV, V and VI was proliferative diabetic retinopathy.

### Quality control

The operation of the same examination and the recording of observation results were fulfilled by the same high-qualification doctor. Moreover, the clinical stage of diabetic retinopathy of all the patients was determined by the same senior doctor from department of ophthalmology.

### Statistical processing

SPSS ver. 21.0 was used for data analysis. Enumeration data were expressed by % and processed by Chi-square test. Difference was considered as statistically significant if P<0.05.

## RESULTS

### Comparison of examination results

Three hundred and seventy-five eyes were diagnosed as diabetic retinopathy by direct ophthalmoscopy (75%). Four hundred and sixty-five eyes were diagnosed as diabetic retinopathy by FFA (93%). The difference between the two diagnostic methods in the detection rate of diabetic retinopathy had statistical significance (P<0.05; Table-I).

**Table-I:** Comparison of FFA and direct ophthalmoscopy results.

*Examination method*		*Direct ophthalmoscopy*	*FFA*	*X^2^*	*P*
Number of cases		500	500		
Without DR		125	35		
Non-proliferative	I	74	94		
	II	88	110		
	III	92	112		
Proliferative	IV	83	92		
	V	28	41		
	VI	10	16		
Rate of lesions		75.00%	93.00%	69.174	0.000

465 eyes were diagnosed as retinopathy by fluorescein fundus angiography, among which, 316 eyes gad non-proliferative diabetic retinopathy (67.96%); the main manifestations of retinopathy in fundus fluorescein angiography included microangioma in macular temple, angiotelectasis and non-perfusion, and the lesions might be located in the optic disk on the side of nose and upper and lower hemal arches. There were 75 eyes of pre-proliferative diabetic retinopathy (16.13%). The main manifestations of pre-proliferative diabetic retinopathy in fundus fluorescein angiography included severe retinal hemorrhage in four quadrants, venous beeding in two quadrants and absence of a wide range of capillary perfusion area; the lesions could cover as wide as four diameters of optic disk in severe cases. There were 149 eyes of proliferative diabetic retinopathy (32.04%); the main manifestation was proliferation of new vessels. There were 135 eyes of diabetic maculopathy (29.03%); the main manifestation was macular edema and macular ischemia. There were 31 eyes of diabetic papillopathy (6.67%).

### Correcting results of direct ophthalmoscopy by FFA

Twenty eyes at stage I of diabetic retinopathy, 22 eyes at the stage II of diabetic retinopathy, 20 eyes at stage III of diabetic retinopathy, 9 eyes at stage IV of diabetic retinopathy, 13 eyes at stage V of diabetic retinopathy and 6 eyes at stage VI were corrected by FFA. One hundred and five eyes were diagnosed without diabetic retinopathy under mydriasis funduscopy; ninety eyes were corrected by FFA (72%).

## DISCUSSION

Screening of diabetic retinopathy aims at identifying cases of retinopathy which need comprehensive treatment. In order to find diabetic retinopathy early, timely adopt symptomatic treatment and reduce the risks of retinopathy induced blindness, the eyes of diabetes patients should be examined regularly. Ophthalmoscopy, a conventional method for screening diabetic retinopathy currently, is featured by simple operation; however, its determination on the early changes of diabetic retinopathy and severity of lesions has distant deficiencies.^10^ Early pathological changes of the retina mainly manifests as microangiopathy.^11^-^13^ Small hemorrhagic spot and microangioma cannot be accurately determined under direct ophthalmoscope. FFA is a novel examination method in clinics currently. Using FFA, the state and blood circulation of retinal vessels can be accurately understood by observing the state of fluorescein in blood circulation.^14^ It can also early discover retinopathy with short course.^15^ Retinopathy manifests as dotted fluorescence, capillary filling defects, papillary ectasia and fluorescence leakage.^16^ Moreover, it can be used for comprehensively understanding the tendency of fundus lesions, observing whether there is increase of microangioma and enlargement of capillary non-perfusion area and the severity of fluorescein leakage, and correctly evaluate the severity of retinopathy and its development tendency.^17^ Zhao XQ considered that FFA could discover earlier pathological changes which cannot be identified by ophthalmoscopy of patients who have suffered from diabetes for less than 5 years,^18^ especially capillary fluorescence leakage. Therefore, patients with diabetes who is not diagnosed as retinopathy by ophthalmoscope can undergo FFA if possible.

In this study, the detection rate of lesions using FFA was much higher than that using direct ophthalmoscope, which was consistent with the research results of Wang XY et al.;^19^ 72% of the eyes which were determined as normal under direct ophthalmoscope were diagnosed having early pathological changes by FFA. Thus it indicated that FFA could identify fundus lesions which were not discovered by direct ophthalmoscope and had a higher accuracy in the early diagnosis of diabetic retinopathy.

Someone considered microangioma as the earliest and most common sign of diabetic retinopathy, while others thought it was capillary fluorescein sodium leakage.^20^,^21^ In this study, the percentage of microangioma was 92.9%, and some patients with diabetic retinopathy only manifested as retinal microangioma, suggesting microangioma was the earliest sign of diabetic retinopathy, which was similar to the research achievements of Lv Peilin.^22^ Capillary leakage will occur, usually after the appearance of microaneurysm, if the permeability of capillary walls changes. In the study, the incidence of capillary leakage was 61.51%. Loss of vision of about 80% of patients with diabetes is induced by diabetic macular edema.^23^ Therefore, early diagnosis is quite important. In this study, the detection rate of macular edema using FFA was 29.03%, suggesting FFA was a reliable method for diagnosing diabetic macular edema. Moreover, FFA could further determine the category of macular edema and its severity to provide a basis for the clinical treatment of diabetic macular edema.

## CONCLUSION

To sum up, FFA is more accurate, precise and reliable in diagnosing diabetic retinopathy. It can evaluate the severity of diabetic retinopathy and related conditions in early stage to guide laser targeted treatment, which buys time for effective treatment. FFA can also protect the vision of patients and relieve the pain of diabetic patients. Thus FFA is of great clinical values.

### Authors’ Contribution

**SHW & YQZ:** Study design, data collection and analysis.

**YQZ, NW & BT:** Manuscript preparation, drafting and revising.

**SHW & BT:** Review and final approval of manuscript.

## References

[1] Ip MS, Domalpally A, Sun JK, Ehrlich JS (2015). Long-term effects of therapy with ranibizumab on diabetic retinopathy severity and baseline risk factors for worsening retinopathy. Ophthalmology.

[2] Sim DA, Keane PA, Rajendram R, Karampelas M, Selvam S, Powner MB (2014). Patterns of peripheral retinal and central macula ischemia in diabetic retinopathy as evaluated by ultra-widefield fluorescein angiography. Am J Ophthalmol.

[3] Looker HC, Nyangoma SO, Cromie DT, Olson JA, Leese GP, Philip S (2013). Predicted impact of extending the screening interval for diabetic retinopathy:the scottish diabetic retinopathy screening programme. Diabetologia.

[4] Feng YQ, Wang RB, Wu XW, Wu SQ, Chen L, Gu Q (2013). Analysis of fluorescence fundus angiography for diabetic retinopathy with retinal vein occlusion. Chin J Chin Ophthalmol.

[5] Yang LX (2015). Observation of the specific manifestations of diabetic retinopathy in fundus fluorescein angiography and its clinical treatment. Chin Health Care Nutr.

[6] Lee CS, Lee AY, Sim DA, Keane PA, Mehta H, Zarranz-Ventura J (2015). Reevaluating the definition of intraretinal microvascular abnormalities and neovascularization elsewhere in diabetic retinopathy using optical coherence tomography and fluorescein angiography. Am J Ophthalmol.

[7] Wang YC, Chen BC, Wang YW, Tie HY, Yang Z (2013). The efficacy of fluorescence fundus angiography in diagnosing diabetic optic neuropathy. Chin J Pract Nervous Dis.

[8] Song J, Pei WX (2016). Clinical observation on laser combined drug and nursing in the treatment of diabetic retinopathy. Laser J.

[9] Team of Fundus Diseases of Ophthalmological Society of Chinese Medical Association (1985). Staging Criteria of Diabetic Retinopathy. Chin J Ophthalmol.

[10] Liao L, Kuang L, Peng S, Yang LL, Zhao Y (2013). The values of fluorescence fundus angiography in diagnosing elderly diabetic retinopathy. Chin J Gerontol.

[11] Wang XH, Xiong QC, Zheng YP, Quan YL, Yu HN (2008). Diagnostic role of FFA in hypoperfusion retinopathy. Int J Ophthalmol.

[12] Ai H, Song HP (2012). Different expression pattern of serum soluble intercellular adhesion molecules-1 and neutrophilic expression of CD18 in patients with diabetic retinopathy. Int J Ophthalmol.

[13] Diaz-Llopis M, Udaondo P, Millán JM, Arevalo JF (2013). Enzymatic vitrectomy for diabetic retinopathy and diabetic macular edema. World J Diabetes.

[14] Moise MM, Benjamin LM, Enoch CY, Igor LP (2013). Mayombian ethnic, vegetables low intake, insulin treatment, diabetic nephropathy and severe diabetic retinopathy are determinants of blindness in diabetic Africans. Int J Ophthalmol.

[15] Xie XW, Xu L, Wang YX, Jonas JB (2008). Prevalence and associated factors of diabetic retinopathy. The Beijing Eye Study 2006. Graefes Arch Clin Exp Ophthalmol.

[16] Sun TLC, Zhou DHR, Kwok SHH, Yu CYI, Wong KYS, Lo MCS (2013). A functional approach to an early diagnosis and early intervention program for pre-school children with special educational needs in Hong Kong. Open J Social Sci.

[17] Varma R, Bressler NM, Doan QV, Gleeson M, Danese M, Bower JK (2014). Prevalence of and risk factors for diabetic macular edema in the United States. JAMA Ophthalmol.

[18] Zhao XQ, Xu L (2010). Diagnostic progress of early diabetic retinopathy. Chin J Clin (Electronic Version).

[19] Wang XY, Chen ZL, Zhong KR, Wu SR, Liao FL (2011). Fundus fluorescein angiography in early diagnosis of diabetic retinopathy application. Chin Mod Med.

[20] Ding S, Zhao S, Wang HX, Wang ZJ, Li YR (2011). Clinical values of non-mydriatic fundus photography in screening diabetic retinopathy. J Pract Diagn Ther.

[21] Zhang ZY, Cao YB, Li XY, Yan XJ, Lou JX, Wang Y (2010). Correlation between diabetic retinopathy and coronary artery stenosis. Prevent Treat Cardio-Cerebr Vasc Dis.

[22] Lv PL, Zhu XP, Shi WH, Yan F (2009). Incidence and clinical conditions of diabetic retinopathy in 565 patients with type 2 DM by initial investigations and early diagnosis. Int J Ophthalmol.

[23] Li LL, Bari GQ, Zhang TZ (2009). Research progress of diagnostic and treatment of diabetic retinopathy. J Med Pharm Chin Minorit.

